# Parental stress and associated factors among parents of preterm neonates admitted at neonatal intensive care unit among selected governmental hospitals Addis Ababa, Ethiopia, 2022. An institution-based cross-sectional study

**DOI:** 10.3389/fpsyt.2024.1377180

**Published:** 2024-08-29

**Authors:** Befkad Derese Tilahun

**Affiliations:** Department of Nursing, College of Health Science, Woldia University, Woldia, Ethiopia

**Keywords:** stress, preterm, neonatal intensive care unit, parent, Ethiopia

## Abstract

**Background:**

The birth of a preterm infant and their subsequent admission to the Neonatal Intensive Care Unit (NICU) is a distressing and challenging experience for parents. The stress experienced by parents can have a significant impact on their mental health, parenting behaviors, and the parent-infant relationship. Recognizing and addressing the stressors faced by parents in the NICU is essential for promoting their well-being and facilitating positive parent-infant interactions.

**Methods:**

A cross-sectional study was conducted at healthcare facilities. The participants for the study were selected using a systematic sampling method, where the sampling interval (K) was calculated for each hospital. Data collection involved the use of a pretested structured questionnaire administered by interviewers. The collected data was analyzed using the Statistical Package for the Social Sciences (SPSS) version 25.0. Simple and multivariable linear regression analyses were performed. Statistical significance was determined using a p-value of less than 0.05.

**Results:**

The accompanying mother (β = 3.094, 95% CI: 3.615, 6.550), length of NICU stay greater than 10 days (β = 5.823, 95% CI: 1.759–9.887), the gestational week between 34 -37 weeks (β = -3.509, 95% CI: -6.358, -.659), parents with college degrees and above (β = -8.667, 95% CI: (-14.111,3.223), mothers who delivered via cesarean section (β = 2.468, 95% CI: -1.822, 4.759), parents without a history of neonatal NICU admission (β = -6.16, 95% CI: -11.69, -.63), and parents whose infant without ventilation (β = -2.755, 95% CI:. -5.492, -.0189) were significantly associated with parental stress.

**Conclusion:**

Parent in this setting revealed high levels of stress during their premature infants’ NICU admission. The gestational week, educational status of the parents, mode of admission, and mode of delivery were found to have significant associations with parental stress. It is important for healthcare providers to recognize and understand the stress experienced by parents when caring for families with preterm infants in the NICU. They should provide support and assistance to parents as they navigate the challenges and stress that come with this situation.

## Introduction

Newborn babies born prematurely or the ones who experience medical complications are admitted to NICUs (1). Having a newborn infant hospitalized in a NICU is an unexpected and stressful event for the family (2). The NICU is a specialized unit that offers medical treatment and nursing care to premature infants or seriously ill newborns. Typically, the crucial process of parent-infant bonding takes place within the initial days after birth, forming the foundation for a lifelong relationship between parents and their children (3). When a baby is born prematurely or faces health risks requiring admission to an NICU, it can impact parent-infant bonding. The combination of the baby being admitted to the NICU and the additional stressors associated with being in an intensive care unit can significantly increase stress levels for the parents (4, 5).

The literature has identified various stressors that may affect the parent of preterm infants in the neonatal intensive care unit (NICU). These stressors include the intricate nature of the NICU’s physical environment, the physical appearance and behaviors of the infants, interactions between staff and parents, and the challenges faced in establishing and maintaining the parent-infant relationship, these specific factors have been recognized as potential sources of stress for the parents of infants admitted to the NICU (6). In addition, a variety of factors, including, socio-economic status, parent perceptions of infant illness, high trait anxiety, and unavailable sources of support (2, 7–9), personality, mental health, family, as well as social and pregnancy related events are likely to contribute to the type and magnitude of stress that parents of preterm neonate experience (10) and, can affect the long-term relationship with their children and their ability to take care of them (11).

Becoming a parent of a premature baby can be an extremely distressing experience. It involves facing the uncertain and critical medical conditions of the infant, witnessing them undergoing invasive treatment with the aid of technology, making crucial decisions about their care, and feeling helpless in their journey. Heightened stress experienced by parents in such situations is a significant risk factor that can disrupt the parent-infant relationship in the early stages of childhood. Consequently, this can contribute to an increased likelihood of long-term difficulties for both children and parents (12).

Nonetheless, while prior research has predominantly concentrated on parental stress related to congenital abnormalities (13), childhood cancer (14), chronic illness in children (15), However, there has been a noticeable lack of comprehensive investigation into the specific area of parental stress caused by having a premature or low-birth-weight infant and currently, little is known about the factors associated with parental stress response to the NICU, especially for parent associated with NICU stress.

Hence, in order to gain a more comprehensive understanding of the impact of the situation, it is essential to identify the underlying causes of stress and evaluate individuals’ coping mechanisms when confronted with challenging circumstances. To achieve this, we employed a standardized and already validated tool to effectively measure the sources, frequency, and intensity of stress experienced in Neonatal Intensive Care Units (NICUs). Through the utilization of this validated instrument, we aimed to provide valuable insights to the research community regarding the factors that elicit stress reactions in parents. While previous investigations in this field have predominantly focused on newborns with critical illnesses, our study deliberately excluded such infants in order to specifically examine the effects of prematurity and separation without the confounding influence of additional trauma from severe illness. The primary objectives of this study were to assess stress levels among parents of premature infants in NICUs and to explore the associated factors.

### Hypothesis

H1: There is a significant association between various factors and parental stress levels among parents of preterm neonates in the NICU.

H0: There is no significant association between the various factors and parental stress levels among parents of preterm neonates admitted at the neonatal intensive care unit.

## Objectives of the study

### General objective

1. To determine the level of stress among parents of preterm infants hospitalized in NICU-selected governmental hospitals in Addis Ababa, Ethiopia, 2022 G.C.
2. To identify factors affecting stress among parents of preterm infants hospitalized in NICU-selected governmental hospitals Addis Ababa, Ethiopia, 2022 G.C.

## Methods

### Study area and period

The research was conducted in a specific government hospital in Addis Ababa, the capital and largest city of Ethiopia. Addis Ababa is situated on a well-watered plateau surrounded by hills and mountains and is centrally located within the country. The city consists of eleven sub-cities and 116 woreda (administrative division), covering an area of 540 square kilometers and hosting a population of over 4,794,000. Addis Ababa’s healthcare system includes eight federal hospitals, six regional hospitals, more than 38 private hospitals, and over 850 private clinics offering various healthcare services. In addition, 86 public health centers provided services to the community.

Participants were recruited from four hospitals in Addis Ababa, Ethiopia. The first hospital was Black Lion Hospital, which houses a Neonatal Intensive Care Unit (NICU) with approximately 33 beds and is considered one of the largest in Ethiopia. The annual admission rate for infants in this NICU is approximately 1700, and it is divided into critical and subcritical rooms for management purposes. The NICU at Black Lion Hospital is part of the federal Ministry of Health care system and has two neonatologists, ten neonatal nurse practitioners, and 45 nurses working in it.

The second hospital included in the study was St. Paulo Hospital, which has an NICU comprising 40 beds and includes 10 functional incubators. This NICU admits approximately 1100 newborns annually and has two neonatologists, 11 neonatal nurse practitioners, and 45 nurses.

The third hospital, the Gandhi Memorial Hospital, is managed by the Addis Ababa Health Bureau and has a 24-bed NICU. The NICU admitted an average of 130 infants per month and had one neonatologist, five neonatal nurse practitioners, and 13 nurses.

The fourth hospital, Yekatit 12 (Abebech Gobena) General Hospital, is under the Addis Ababa Health Bureau. Its NICU has an annual admission rate of 900 infants and consists of 36 beds, including nine functional incubators. The NICU had one neonatologist, four neonatal nurse practitioners, and 31 nurses.

### Study period

The study was conducted over a period of one month, from March 3 to March 30, 2022.

### Study design

A facility-based cross-sectional study was conducted to assess the level of stress associated with parents of preterm neonates hospitalized in NICU-selected governmental hospitals in Addis Ababa, Ethiopia, in 2022 G.C.

### Source population

All parents of preterm neonates hospitalized in NICU-selected governmental hospitals in Addis Ababa, Ethiopia, in 2022.

### Study population

All selected parents of preterm neonates hospitalized in NICU-selected governmental hospitals in Addis Ababa, Ethiopia, in 2022.

### Inclusion and exclusion criteria

Inclusion criteria: was the mother and father of a premature infant of 32–37 (we included ^36 weeks +6 days^ but we used the upper border 37weeks for classification purpose) of gestational age, hospitalized at the NICU?

All the parents who signed the informed consent and parents whose children had been in the NICU or the PICU for at least 2 days.

### Sample size determination and sampling procedures

The size of the sample was determined using the formula for a single population mean. Based on previous research and a reference study conducted remotely, the mean score for parental uncertainty was found to be 78.8, with a standard deviation of 18.8 (16) To ensure an acceptable level of accuracy and account for potential discrepancies, a maximum discrepancy of 2.2 between the sample size and the underlying population was considered. Taking these factors into account, the sample size was calculated. Additionally, a pilot study was conducted, and various recently published pieces of literature were reviewed, all of which gave a smaller sample size. Therefore, the mean and standard deviation mentioned earlier were used to determine the sample size. Considering a 10% non-response rate, the total sample size was determined to be (277 + 28) = 305.

### Sampling procedure

A total of thirteen governmental hospitals in Addis Ababa were available for selection, and four hospitals were chosen using a simple random sampling method. The hospitals included in the study were Tikur Anbessa Specialized Hospital, Gandhi Memorial Hospital, St. Paul’s Hospital Millennium Medical College, and Yikatit 12 (Abebech Gobena) Hospital.

**Figure 1:**
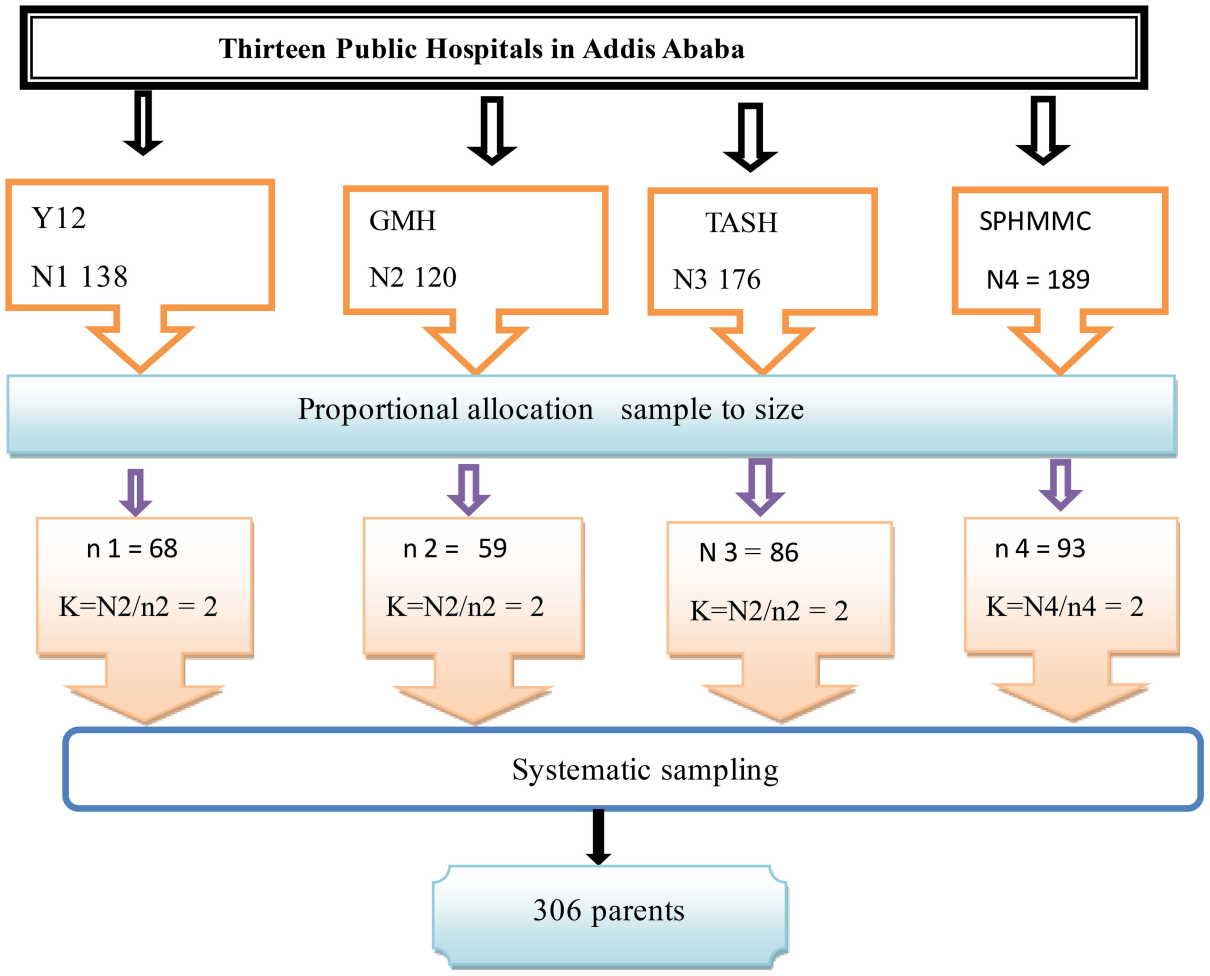
Schematic presentation of the sampling technique. Y12, Yikatit 12 hospital; TASH: Tikur Anbessa Specialized Hospital; GMH, Gandhi Memorial Hospital; SPHMMC, St. Paulo’s hospital millilumen medical college.

To collect data during the study period at each hospital, a systematic random sampling technique was employed, utilizing the mother’s medical registration logbook. The value of K, calculated as N/n, where N represents the total number of mothers in the hospitals (623), and n represents the sample size (305), was found to be 2. This means that every two parents were interviewed for data collection purposes (**Figure 1**).

### Study variables

#### Dependent variable

Parental stress.

#### Independent variables

Sociodemographic factors: Age, sex, education level, economic status, education status, family arrangement, residence, mode of delivery, mode of admission, previous history of neonatal NICU admission, and previous history of neonatal morbidity and mortality.

Biophysical characteristics of the preterm neonate: Low birth weight, low gestational age, pathologic condition, ventilation care.

Health institution-related factors: The presence of monitors and equipment, the other sick babies in the room, having a machine breathe (ventilator), and the constant noise of monitors and alarms compromise privacy and hospital costs.

## Data collection instruments, methods, and procedures

### Data collection instruments

The data collection process involved the use of a pretested, structured interviewer-administered questionnaire. Additionally, the mother’s chart, with the guidance of data collectors, was utilized to gather relevant information. The interviewers reviewed medical records to retrieve data on ventilation care (yes or no), pathological condition of the preterm baby, number of pregnancies, and mode of delivery (vaginal, instrumental, or cesarean). Information on the infant’s gender, birth weight, and gestational age was also recorded.

To assess the parent’s perception of stressors within the Neonatal Intensive Care Unit (NICU), the researchers utilized the Parental Stressor Scale: Neonatal Intensive Care Unit (PSS-NICU), which was developed by Miles and Funk (14). This scale was included in the questionnaire to measure the levels of stress experienced by parents. In this study, the Italian version of the Parental Stressor Scale: Neonatal Intensive Care Unit (PSS: NICU) by Montirosso was used to assess stress levels in both mothers and fathers (17). The PSS: NICU is a self-report measure consisting of 26 items that assess parental stress in three dimensions during their stay in the Neonatal Intensive Care Unit (NICU). These dimensions include Sights and Sounds (SS), which captures stress related to the physical environment of the NICU (6 items); Infant Behavior and Appearance (IBA), which measures stress arising from the appearance and behavior of the infants (13 items); and Parental Role Alteration, which examines stress associated with changes in expected parental roles and the postponement of actual parental care (7 items). Parents are required to rate each item on a five-point Likert scale, ranging from ‘not stressful’ to ‘extremely stressful’.

To compute the overall stress level for each subscale, the sum of scores for the items within the subscale is divided by the number of items. The subscale scores range from 1 to 5, with lower scores indicating a lower perceived level of parental stress. This questionnaire has demonstrated good psychometric properties, including test-retest reliability ranging from 0.69 for subscales to 0.87 for the total scale and internal consistency ranging from 0.73 to 0.92 for subscales and 0.89 to 0.94 for the total scale. In this particular study, the PSS: NICU tool exhibited excellent psychometric properties, with Cronbach’s α reliability coefficients ranging from 0.905 to 0.922. Mean scores were calculated for each subscale, as well as the mean overall scores.

### Operational definition and terms

Parent: is a caregiver of the offspring in their neonates (biological mother or father of the neonate).

Stress: measured by using the PSS: NICU instrument can be scored either by calculating the frequencies of stressful experiences, and it has 26 items, with each item having a five-point response scale like “1: Not at all stressful 2-A little stressful 3-Moderate stress 4-Very stressful 5: Extreme stress.”.

Parental stress: refers to the psychological and emotional strain experienced by parents due to the unique challenges and uncertainties associated with the medical care and development of their premature baby. It encompasses feelings of worry, anxiety, helplessness, and fear that arise from factors such as the infant’s fragile health, medical interventions, extended hospitalization, separation from the baby, limited control over the caregiving process, and uncertainty about the infant’s long-term outcomes.

Low multiparity (LM): a woman with 2–4 deliveries after 28 weeks of gestation (18).

Grand multiparity (GM): a woman with ≥5 births after 28 weeks of gestation (18).

Small family size: family size less than 5 (19).

Medium family size: family size greater than 5 (19).

### Data collection procedure

Data collection was performed by a team consisting of four nurses holding bachelor’s degrees, while two master’s degree students were designated as supervisors for the data collection process. Prior to the commencement of data collection, two days of training were provided to the data collectors and supervisors by the principal investigator. This training covered the appropriate approach to study participants and the proper method of completing the questionnaires.

The principal investigator oversaw the entire data collection process to ensure its smooth execution. The selected participants were informed by the data collectors about the importance and relevance of the study, and they were invited to take part in the research.

### Data quality control

To ensure the quality of the data, meticulous measures were implemented at various stages of the study. In all study areas, a team of four data collectors and two supervisors who were proficient in English and Amharic languages were recruited. Preference was given to individuals with prior experience in similar field surveys.

To ensure effective and high-quality data collection, the selected data collectors underwent a two-day intensive training session conducted by the principal investigator. The training aimed to equip them with the necessary skills and knowledge to achieve the study’s objectives.

Before commencing the actual data collection, a pre-test was conducted using 5% of the questionnaires. This pre-test involved parents of preterm neonates in Debre Birhan town. Based on the findings from the pre-test, any necessary modifications were made to the questionnaire to enhance its clarity and effectiveness.

### Data processing and analysis

The collected data underwent a series of steps for coding and cleaning. After coding, the data was entered into Epi-data version 4.6.2 to identify outliers, missing values, and inconsistencies. Subsequently, the data was exported into SPSS version 25.0 for further analysis.

Descriptive statistics, such as frequency, percentage, mean, and standard deviation, were used to summarize the socio-demographic characteristics of the sample. The Pearson correlation coefficient was employed to determine the relationships between continuous variables. All assumptions for linear regression were verified. The scatter plot was examined to confirm linearity between the dependent and independent variables. The histogram and Q-Q plot were utilized to assess multivariate normality. The scatter plot was employed to evaluate homoscedasticity, indicating similar residuals around the regression line. The Variance Inflation Factor (VIF) was calculated to detect multicollinearity, and all variables had a value below 7, indicating the absence of multicollinearity in the final model. Outliers were assessed using scatter plots, and it was found that all assumptions were met.

Initially, simple linear regression was conducted to select candidate variables for the multivariable linear regression analysis. Variables with a p-value ≤ 0.25 in the simple linear regression analysis, as well as variables deemed important by the researcher, were considered as candidate variables for the multivariable linear regression. The results showed that skewness ranged between −2.49 and 2.03. The values of kurtosis ranged between −1.96 and 4.41. Considering skewness and kurtosis together the results indicated that the distributions were close to expected values under normality. After performing the multivariable linear regression analysis, variables with p-values < 0.05 were regarded as having a statistically significant association with the dependent variable. The strength of the association between the independent and dependent variables was assessed using an unstandardized β with a 95% confidence interval.

## Results

### Socio-demographic characteristics of the parents

Out of the total of 306 subjects recruited for the study, three parents were unable to complete the interview, resulting in a final sample size of 303 participants with complete data. This yielded a response rate of 99.01%. Among the participants, there were 259 mothers and 44 fathers of preterm infants (PTI) who were hospitalized in four NICU hospitals in Addis Ababa, Ethiopia.

Within the sample, the majority of parents, 71.9% (218 individuals), were between the ages of 25 and 35 years. Regarding educational attainment, 39.6% (120 individuals) had completed college or above, 35.0% (106 individuals) were high school graduates, and 19.1% (58 individuals) had completed primary school. The majority of participants were married, accounting for 86.5% (262 individuals) of the sample.

In terms of occupation, 41.3% of the parents were housewives, while 28.7% were employed. Furthermore, 60.4% of the parents had only one child (**Table 1**).

**Table 1:** -Socio demographic characteristics of the parent preterm neonate at NICU of selected governmental hospitals Addis Ababa, Ethiopia 2022 (N=303).

Parent demographic data	Categories	Frequency	Percent (%)
***Gender of the respondent***	Female Male	259 44	85.5 14.5
***Age of the parent***	<19 19–25 25–35 >35	5 29 218 51	1.7 9.6 71.9 16.8
***Level of education***	College & above Secondary Primary Unable to read and write	120 106 58 19	39.6 35.0 19.1 6.3
***Marital status***	Married Unmarried	262 41	86.5 13.8
***Usual residence***	Addis Ababa Out of Addis Ababa	229 74	75.6 24.4
***Mode of admission***	Referred from other facility Self-referral	127 176	41.9 58.1
***Previous history of child mortality and morbidity***	Yes No	62 241	20.5 79.5
***Previous history with newborn admission to NICU***	Yes No	31 272	10.2 89.8
***Family size***	Small family size Medium family size	196 105	64.7 34.7
***Number of parities*** ***Mode of delivery*** ***Occupational statues*** ***/***	Low multiparity Grand parity Vaginal/instrumental Caesarean Civil servant House wife Student Private business	293 10 243 61 87 169 17 30	96.7 4.3 79.9 20.1 28.6 55.6 5.6 9.9

### Biophysical characteristics of the infants

In terms of the infants, 55.8% (169 infants) were female, and 69.6% (211 infants) were born between 32 - 34 weeks of gestation. The majority of infants, 85.2% (259 infants), had a birth weight above 2000 grams.

Furthermore, 68.8% of the infants included in the study had a hospital stay in the neonatal intensive care unit (NICU) of less than 5 days. Among the infants, 53.9% (164 infants) were admitted due to respiratory distress syndrome (RDS), while 16.8% (51 infants) were diagnosed with both RDS and sepsis. Additionally, 69.6% (211 infants) received respiratory care.

Most of the neonates, accounting for 79.5% (243 infants), were delivered through vaginal or instrumental delivery methods (**Table 2**).

**Table 2:** Biophysical characteristics of the preterm neonate at NICU of selected governmental hospitals Addis Ababa, Ethiopia 2022 (N=303) .

Biophysical characteristics of the preterm infants		Frequency	Percent
***Sex of the infant***	Female Male	169 134	55.8 44.2
***Pathologies***	Other Preterm only RDS + Sepsis (RDS) Sepsis	41 17 51 164 30	13.5 5.6 16.8 53.9 9.9
***Ventilation care***	Yes No	211 92	69.9 30.1
***Gestational week***	Moderate preterm Late preterm	211 92	69.9 30.1
***Weight of the infant***	Very low birth weight Low birth weight Normal birth weigh	29 259 15	9.5 85.2 4.9
***Length of NICU stay in days***	<5 5–10 >10	209 59 35	68.8 19.4 11.8

### Stressful situations in a NICU

Drawing from the existing literature and the NICU stress framework, parents’ reported sources of stress were categorized into two constructs: the baby’s appearance and behavior, and the environmental sights and sounds within the NICU.

### Baby’s appearance and behavior

The average stress score related to the baby’s appearance and behavior reported by the study participants was 43.79 (SD = ± 0.414) out of a total of 13 items. Among all respondents, 130 (42.90%) participants expressed being significantly stressed when their baby appeared sad. Additionally, 121 (39.93%) participants reported feeling very stressed when their baby exhibited abnormal breathing patterns.

In contrast, only 4 (1.32%) respondents indicated no stress at all when their baby did not cry like other babies, while 10 (3.30%) participants reported no stress when their baby had a smaller size. Moreover, 40 (13.20%) participants experienced extreme stress when their baby required an intravenous feed line (**Table 3**).

**Table 3:** Parents’ stress of how their babies appeared and behaved at NICU of selected governmental hospitals Addis Ababa, Ethiopia 2022 (N=303).

Items	Not at all stressful	A little stressful	Moderate stress	Very stressful	Extreme stress
Baby looked sad	5(1.65)	41(13.53)	69(22.77)	130(42.90)	58(19.14)
limp and weak	13(4.29)	58(19.14)	92(30.36)	105(34.65)	35(11.55)
Needles and tubes inserted	6(1.98)	37(12.21)	94(31.020	109(35.97)	57(18.81)
Abnormal breathing patterns	8(2.64)	34(11.22)	104(34.32)	121(39.93)	36(11.88)
pain	2(0.66)	63(20.79)	96(31.68)	100(33.00)	42(13.86)
Intravenous feed line or tube	3(0.99)	28(9.240	93(30.69)	139(45.87)	40(13.20)
Baby’s unusual color	9(2.97)	54(17.82)	89(29.37)	116(38.28)	35(11.55)
Tubes and equipment	4(1.32)	37(12.21)	85(28.05)	125(41.25)	52(17.16)
Jerky or restless movements	24(7.92)	82(27.06)	106(34.98)	72(23.76)	19(6.27)
Wrinkled appearance	31(10.23)	83(27.39)	73(24.09)	95(31.35)	21(6.93)
Bruises, cuts, or incisions	32(10.56)	84(27.72)	96(31.68)	72(23.76)	19(6.27)
Do not cry like other babies	10(3.30)	53(17.49)	69(22.77)	134(44.22)	37(12.21)
having a small size baby	4(1.32)	28(9.24)	88(29.04)	135(44.55)	48(15.84)

### Sights and sounds in the NICU

The average stress score regarding the sights and sounds in the NICU, as reported by the study participants, was 19.90 (SD = ± 0.202) out of a total of 6 items. The behavior that was perceived as the most stressful was when a baby stopped breathing, with 117 (38.61%) parents reporting extreme stress responses to this situation.

Additionally, the presence of other sick babies in the room was reported as a stressor by 93 (30.69%) parents, with 61 (20.13%) of them experiencing extreme stress reactions. The presence of monitors and equipment also elicited extreme stress responses from 61 (20.13%) parents (**Table 4**).

**Table 4:** Parents’ stress of sights and sounds in the NICU at NICU of selected governmental hospitals Addis Ababa, Ethiopia 2022 (N=303).

Items	Not at all stressful	A little stressful	Moderate stress	Very stressful	Extreme stress
The presence of monitors and equipment	14(4.62)	40(13.20)	73(24.09)	115(37.950	61(20.130
The constant noise of monitors and alarms	5(1.650	33(10.89)	98(32.34)	148(48.84)	19(6.27)
The sudden noise of monitors and alarms	29(0.66)	46(15.18)	101(33.33)	128(42.24)	26(8.58)
The other sick babies in the room	4(1.32)	23(7.59)	87(28.71)	126(41.58)	63(20.79)
The large number of people working in the unit	21(6.93)	55(18.15)	119(39.27)	87(28.71)	21(6.93)
Having a machine breathe for my baby	18(5.94)	48(15.84)	96(31.68)	24(7.92)	117(38.61)

### Parental role alteration

The average score for parental role alteration reported by the study participants was 21.27 (SD = ± 0.218) out of a total of 7 items. The most common parental role alteration was experienced when parents were unable to hold their baby when they desired, with 39 (12.87%) participants reporting this as a significant stressor.

A majority of the participants, 146 (48.18%), reported parental role alteration when they felt helpless and unable to protect or assert themselves during their stay in the facility. Out of all the respondents, 127 (41.91%) expressed feeling very stressed when they were unable to hold their baby when they wanted. Only 4 (1.32%) of the respondents reported no stress at all when they couldn’t provide care (**Table 5**).

**Table 5:** Parents’ perceptions of parental role adjustment at NICU of selected governmental hospitals Addis Ababa, Ethiopia 2022 (N=303).

Items	Not at all stressful	A little stressful	Moderate stress	Very stressful	Extreme stress
cannot feed	4(1.32)	28(9.24)	88(29.04)	135(44.55)	48(15.84)
cannot provide care	4(1.32)	29(9.57)	98(32.34)	137(45.21)	35(11.55)
Cannot hold baby when I want	3(0.99)	31(10.23)	103(33.99)	127(41.91)	39(12.87)
No time to be alone with baby	1193.63)	32(10.56)	104(34.32)	122(40.26)	34(11.22)
Feel helpless and cannot protect	3(0.99)	34(11.22)	94(31.02)	146(48.18)	26(8.58)
separation	1193.63)	32(10.56)	104(34.32)	122(40.26)	34(11.22)
Feeling helpless in terms of assistance	21(6.93)	46(15.18)	129(42.57)	94(31.02)	13(4.29)

When examining the mean scores of the PSS: NICU subscales, the highest mean score belongs to the subscale of the infant’s appearance and behaviors (43.79), followed by the parents’ role alterations mean score (21.27) and then the sights and sounds subscale mean score (19.90). Based on the results, it was determined that the area that causes the most stress for parents is their infant’s appearance and behaviors (**Table 6**).

**Table 6:** Distribution of percentage mean score of parental stress scale at NICU of selected governmental hospitals Addis Ababa, Ethiopia 2022 (N=303).

Variable	Mean	Std. errs.	[95% conf. interval]
Infant behavior appearance	**43.79538**	.4144953	42.97971 - 44.61104
Parental role alteration	**21.27063**	.2184776	20.8407 - 21.70056
Sight sound	**19.90099**	.2025732	19.50236 - 20.29962

Bold values is the Mean value/ mean result of our study.

### Factors affect parental stress

A multiple linear regression analysis was conducted to examine the impact of independent variables on parental stress among parents of preterm neonates in the NICU. Variables with a p-value less than 0.25 and considered significant by the researcher were included in the final regression model for analysis.

Therefore, in the multiple linear regression analysis, variables with a p-value less than 0.25 were included. These variables encompassed the sex of the parent, mode of admission, gestational week, pathology of the preterm neonate, ventilation care of the preterm neonate, residency of the parent, length of NICU stay, educational status, history of neonatal morbidity and mortality, history of neonatal NICU admission, and occupation status of the parent. All of these variables were found to be significant factors influencing parental stress in the multiple linear regression analysis.

In this particular study, preterm was found to have a significant association with the outcome variable. When controlling for the effects of other factors, it was observed that parental stress among parents of preterm neonates decreased by -2.755 in cases where the preterm baby did not receive ventilation care (P = 0.0048: 95% CI: -5.492, -0.0189).

Furthermore, when all other variables in the model were held constant, it was found that parents without a history of neonatal morbidity and mortality had a significant association with parental stress. In these cases, parental uncertainty decreased by -3.440 (P = 0.041: 95% CI: -6.735, -0.146) for parents without a history of neonatal morbidity and mortality, as well as a history of NICU admission.

This study’s results showed that parents of preterm neonates whose gestational weeks were between (34–37) or late gestation decreased parental stress by -3.509 (P =. 0.016: 95% CI of β: -6.358, -.659).

However, it should be noted that there was a positive correlation between the length of NICU stay and parental stress. Specifically, neonates who stayed in the NICU for more than ten days experienced an increase in parental uncertainty by 5.823 (P = 0.005: 95% CI of β: 1.759–9.887) compared to neonates who had a NICU stay of less than five days. This association held true when all other variables in the model were held constant.

Similarly, the mode of delivery, specifically Caesarean section, was found to increase parental stress by 2.468 (P = 0.003: 95% CI of β: 1.822, 4.759) when compared to spontaneous vaginal delivery (SVD). This effect was observed while controlling for the influence of other variables in the model (**Table 7**).

**Table 7:** Bivariate and multivariate linear regression analysis for factors affecting parental stress of preterm infants at NICU of selected governmental hospital Addis Ababa,20222.

Variables	Categories	Crud β (95% CI	95%CI for β	Adjusted β (95% CI)	95%CI for β	p-value
**Mode of admission**	Self-referral Referred	7.48	4.712- 10.258	1.956	-.800–4.71	0.163
**Ventilation care**	Yes No	-5.565	-8.669, -2.461	-2.755	-5.492, -.0189	0.0048
**Length of NICU stay**	<5 5–10 >10	6.790 10.221	3.287- 10.293 5.881- 14.561	4.002 5.823	.800–7.204 1.759–9.887	0.014 0.005
**Educational status**	Unable to read and write Primary Secondary College & above	-2.058 -1.647 -13.846	-7.868, 3.750 -7.123, 3.828 -19.273, 8.419	-1.995 -.533 -8.667	-7.479, 3.487 -5.762, 4.696 -14.111,3.223	0.474 0.841 0.002
**Gestational weeks**	32–34 34–37	-9.095	-12.048, 6.142	-3.509	-6.358, -.659	0.001
**History of neonatal morbidity and mortality**	Yes No	-7.767	-11.203, 4.332	-3.440	-6.735, -.146	0.041
**Residency**	Addis Ababa Out of Addis Ababa	8.965	5.794, 12.137	.761	-2.678, 4.200	0.663
**Mode of delivery**	Vaginal/instrumental Caesarean	2.658	-.902, 6.220	2.468	1.822, 4.759	0.003
**Sex of the parent**	Male Female	2.564	-6.615, 1.486	3.094	3.615, 6.550	0.002

## Discussion

The aim of our study was to assess the level of parental stress and identify the factors associated with it among parents of preterm infants (PTI) receiving care in the neonatal intensive care unit (NICU). We collected data on the parents’ stress levels related to their neonates’ hospitalization and investigated the various factors that contribute to parental stress in this context.

In this study, it was observed that mothers were approximately three times more likely to experience stress compared to fathers. This finding aligns with previous research indicating that mothers tend to experience higher levels of stress than fathers (20). However, this result contradicts the findings reported by Luis et al., which suggests a discrepancy in the findings between the two studies (4, 9, 21). Likewise, another study reported no significant distinction between mothers and fathers in terms of stress levels as measured by the PPS: NICU (22). This inconsistency can be attributed to the fact that mothers often deviate from their regular routines and spend extended hours in the NICU, while fathers continue to experience concerns about their infants’ vulnerability and mortality. Moreover, mothers tend to experience a greater sense of guilt than fathers regarding their premature child’s health issues. Additionally, mothers and fathers may employ different coping strategies to manage parental stress. Research suggests that mothers often rely on seeking social support, self-disclosure, and emotion-focused coping strategies. Fathers, on the other hand, may engage in problem-solving approaches and instrumental support seeking. These differences can influence the perception and management of stress.

In this study, the age of parents and their marital status did not demonstrate a significant correlation with levels of parental stress. This finding is consistent with previous research conducted on parents of children with chromosomal conditions, where similar associations were observed (20, 21, 23). The lack of significance in these associations could potentially be attributed to the limited variability in the age group, as a majority (71.9%) of the parents in this study fell within the 25–35 age range. Similarly, the high percentage (86.5%) of married parents in the sample may have contributed to the non-significant findings.

The findings of this study revealed a significant correlation between parental education level and parental stress. Specifically, parents with a college degree or higher education demonstrated a significant negative association with parental uncertainty compared to parents with no formal education (14, 23, 24). Similar results were documented in studies conducted in New Zealand, which indicated a negative correlation between educational level and parental stress. These studies found that parents with higher educational attainment reported lower levels of stress compared to parents with less than a secondary education (25), however this result is inconstant with study (26). This could be attributed to the fact that education serves as a basis for personal knowledge and enables individuals to comprehend health-related situations. Furthermore, it can enhance parents’ capacity to access pertinent information and engage in effective problem-solving, including self-adaptation. The reason behind this could be that well-educated individuals have a deeper understanding of preterm education compared to those with lower levels of education (27). Additionally, educated parents are more likely to access information from various sources such as books, leaflets, and magazines, and they may have greater exposure to preterm education through mass media compared to parents with lower levels of education.

Parents of preterm infants delivered between 34–37 weeks gestation reported significantly lower levels of parental stress compared to parents whose infants were delivered between 32–34 weeks gestation. This finding is consistent with previous studies that have demonstrated a significant negative association between ‘Weeks of gestation’ and parental uncertainty (p < 0.001) (20, 21, 28, 29). This observation may be explained by the fact that in this study, only a small percentage (6.3%) of parents had limited literacy skills, indicating that most parents had a good understanding of early and late preterm delivery. As they approached term, parents may have perceived it as a safer period for their infants, leading to lower levels of stress (5, 30). Conversely, if parents had a longer hospital stay, they may have perceived their infant’s condition as more serious and incurable, ultimately contributing to higher levels of parental uncertainty. It is also possible that infants born at a younger gestational age required a longer duration of stay in the NICU, which could have resulted in increased paternal uncertainty.

Likewise, parents whose neonates did not require ventilation care exhibited a significant negative association with parental stress compared to parents whose neonates received ventilation care. This finding aligns with a previous study that reported similar results (5, 30). This finding may be attributed to the parents’ expressed desire for all possible measures to be taken for their infants in the delivery room. Parents whose infants required respiratory support and were connected to respiratory equipment experienced elevated levels of stress in comparison to parents whose infants were breathing normally (30, 31). This presented a challenge because, despite the infant receiving ventilation support and exhibiting signs of breathing, parents perceived these infants as having a lower likelihood of survival.

Parents who did not have a history of neonatal mortality or morbidity, experienced a significant reduction in parental stress as compared to parents who had a history of neonatal, morbidity and mortality. This finding aligns with previous research conducted on the subject (20, 21, 32) This could be attributed to negative experiences in previous NICU stays and also parents with a history of neonatal mortality or morbidity may have heightened concerns about the long-term health and development of their newborn. The experience of previous loss or complications can lead to heightened anxiety and worry about their baby’s future, contributing to increased parental stress.

Parent whose mode of delivery were caesarean section nearly three times more stressed than its counterpart. This is in line with the studies (4, 21, 29). This could be attributed to several factors specific to preterm births and caesarean section. Firstly, preterm births are often unexpected and can bring about concerns regarding the health and well-being of the infant. The added complexity of a caesarean section delivery, with its associated surgical procedure and potential complications, may further contribute to parental stress. The recovery process for parents after a caesarean section, along with the need for additional medical interventions and care for the preterm infant, can also add to the overall stress experienced by these parents.

Parent whose neonate’s had long duration of hospital admission five times increase parental stress than its counterpart. This finding consistent with the findings (4, 21, 30). Parents of preterm infants who experience a long duration of hospital admission often face increased levels of parental stress. This can be attributed to various factors associated with an extended hospital stay. Firstly, the prolonged separation from their newborn during the hospitalization period can cause significant emotional distress and anxiety for parents. Being unable to have their infant at home and participate in typical parenting activities can be challenging and emotionally draining (30). Furthermore, the complex medical care required during the extended hospitalization, including frequent medical procedures and interventions, can add to parents’ stress. The constant monitoring, consultations with healthcare professionals, and uncertainty surrounding the infant’s progress can be overwhelming for parents. Overall, the combination of emotional separation, concerns about the infant’s well-being, complex medical care, and financial implications contribute to increased parental stress in cases where preterm infants have a long duration of hospital admission.

### Limitations of the study

The study has several methodological limitations that should be considered when interpreting the findings. Firstly, the study was conducted in selected governmental hospitals in Addis Ababa, Ethiopia, which may limit the generalizability of the findings to other settings or populations. The results may not be representative of parents of preterm neonates in other regions or non-governmental hospitals. Additionally, the study employed a cross-sectional design, capturing data at a single point in time, which limits the ability to establish causal relationships and understand changes in uncertainty levels over time. A longitudinal design would have provided a better understanding of the dynamics and factors influencing parental stress. The study relied solely on self-reporting by parents, which may introduce recall bias or social desirability bias. Parents may not accurately recall or report their levels of stress, leading to potential measurement errors. Our study found that we did not provide detailed elaboration on the specific confounders and effect modifiers considered in our analysis. Future research should aim to investigate and incorporate relevant confounders and explore potential effect modifiers to obtain a more comprehensive understanding of the relationship under study. Finally, we do not know if or how previous experiences of stress, obstetric comorbidities, medical comorbidities in the parents, and psychiatric comorbidities might affect parents’ experiences of stress during hospitalization, and whether the observed differences in our study are persistent over time is unknown as our study did not include any follow-up data. To gain a better understanding of the factors contributing to parental stress, future studies should include assessments of psychiatric and obstetric comorbidities and collect follow-up data from parents of preterm newborns. This will allow for a clearer understanding of the factors influencing parental stress.

## Conclusion

This study established an initial understanding of the level of parental stress in the specific study region. The findings indicated that parental stress was significantly associated with factors such as the educational status of the parent, the respondent’s gender, the mode of admission, the use of ventilation care, and the length of stay in the NICU.

## Recommendation

The results of this study provide important information for health professionals who are working at the NICU in Ethiopia. Parents should be provided with assistance to improve their self-esteem and confidence in neonatal intensive care units. Encouraging parents to visit their babies, teaching them how to care their babies and including them in decisions constitute foundations of this process. Establishing a welcoming atmosphere by engaging parents in the care of their infants, providing educational resources, and fostering effective communication aids parents in developing a sense of ease in their role as caregivers. Strategize targeted measures to help parents cope with the stress of having preterm babies with low GA and needing respiratory support in the NICU. Communicate information about preterm birth to parents using language they understand and at an appropriate educational level, ensuring clear comprehension of the situation. In addition, researchers recommend that health care providers should be supported parents with psycho-emotional problems, strengthen parents–healthcare workers’ interaction, and scale up neonatal intensive care unit services to the primary health care centers. Moreover, research is required to gain a clearer understanding of the specific risk factors that contribute to heightened parental stress and negative emotional states associated with premature birth.

## Data availability statement

The original contributions presented in the study are included in the article/supplementary material, further inquiries can be directed to the corresponding author/s.

## Ethics statement

The studies involving humans were approved by Addis Ababa institutional review board. The studies were conducted in accordance with the local legislation and institutional requirements. The participants provided their written informed consent to participate in this study.

## Author contributions

BT: Conceptualization, Data curation, Formal analysis, Investigation, Methodology, Resources, Software, Supervision, Visualization, Writing – original draft, Writing – review & editing.
